# The development of an implementation framework for service-learning during the undergraduate nursing programme in the Western Cape Province

**DOI:** 10.4102/curationis.v38i2.1563

**Published:** 2015-11-13

**Authors:** Hester Julie

**Affiliations:** 1 School of Nursing, University of the Western Cape, South Africa

## Abstract

**Background:**

Service-learning (SL) is a contested field of knowledge and issues of sustainability and scholarship have been raised about it. The South African Higher Education Quality Committee (HEQC) has provided policy documents to guide higher education institutions (HEIs) in the facilitation of SL institutionalisation in their academic programmes. An implementation framework was therefore needed to institutionalise the necessary epistemological shifts advocated in the national SL policy guidelines.

**Objectives:**

This article is based on the findings of a doctoral thesis that aimed at developing an SL implementation framework for the School of Nursing (SoN) at the University of the Western Cape (UWC).

**Method:**

Mixed methods were used during the first four phases of the design and development intervention research model developed by Rothman and Thomas.

**Results:**

The SL implementation framework that was developed during Phase 3 specified the intervention elements to address the gaps that had been identified by the core findings of Phases 1 and 2. Four intervention elements were specified for the SL implementation framework. The first intervention element focused on the assessment of readiness for SL institutionalisation. The development of SL capacity and SL scholarship was regarded as the pivotal intervention element for three of the elements: the development of a contextual SL definition, an SL pedagogical model, and a monitoring and evaluation system for SL institutionalisation.

**Conclusion:**

The SL implementation framework satisfies the goals of SL institutionalisation, namely to develop a common language and a set of principles to guide practice, and to ensure the allocation of resources in order to facilitate the SL teaching methodology. The contextualised SL definition that was formulated for the SoN contributes to the SL operationalisation discourse at the HEI.

## Introduction

Service-learning (SL) is a relatively new field in the social and educational landscapes (HEQC 2006b:138; Le Grange 2007:3). Butin’s (2006a:1) claim that SL is actively supported by governments because of the inherent potential that SL has to contribute to the societal transformation agendas of governments is confirmed by other scholars (Bender 2008:84; Erasmus 2007:110; Weerts & Sandman 2010:632–633). SL scholars and practitioners, however, have raised concerns about SL issues related to sustainability (Brukardt *et al.* 2004:4), transformative practice (Butin 2006b:478) and authentic institutional commitment (Brukardt *et al.* 2004:17). Consequently, scholars such as Bringle and Hatcher (2000:288) and Butin (2006b:473) argue for the institutionalisation of SL within a scholarly framework.

The institutionalisation of SL in the academic programmes of South African HEIs is guided by the policy documents of the Higher Education Quality Committee (HEQC 2004a:7–8, b:7, 19; 2006a:7–9, b:138–146). Authentic deliberations and open discourse about the status quo of civic engagement (CE) and SL in the South African higher education sector (Smith-Tolken & Williams 2011:9–10) reveal that although the CE and SL policies of South Africa are highly regarded (Hall 2010:24), scholars remain concerned about their implementation at multiple levels (Albertyn & Daniels 2009:410). The critique relates mainly to the disjuncture between the SL policy intention and implementation at institutional level. The Council on Higher Education's (CHE) rebuttal to the SL policy criticism is that it is incumbent on HEIs to institute the changes proposed by national policy (HEQC 2006b:12).

### Problem statement

The latest institutional audit by the HEQC suggests that the strategic objectives of the UWC in relation to CE and SL implementation strategies need to be reviewed (CHE 2008:19). Likewise, the SoN at the UWC has had an obligation to formalise a framework for institutionalising SL in its nursing programme in order to encapsulate the mission of the UWC as an engaged institution and to facilitate the necessary epistemological amendments (Julie 2014b:1832) advocated in the national SL policy guidelines (HEQC 2006b:138–146).

#### Objective

The aim was to develop an SL implementation framework for the SoN at the UWC.

#### Definition of key concepts

**Engagement:** The partnership between the knowledge and resources of a university and the expertise of the public and private sectors enriches scholarship, research and creative activity; enhances the curriculum, teaching and learning; prepares educated, engaged citizens; strengthens democratic values and civic responsibility; addresses critical societal issues, and contributes to the public good (McNall *et al.* 2009:318).

**Framework** is defined as a logical grouping of related concepts usually created to amalgamate several different aspects that are relevant to a complex situation, such as a practice setting or an educational programme (Chinn & Kramer 2004:60).

**Service-learning (SL)** has been conceptualised as an engaged pedagogy that integrates theory with relevant community service projects. The SL assignments and group discussions have been designed to facilitate a more reflective approach towards greater integration of the contents of the psychiatric mental health nursing and gender-based violence modules with social responsiveness within nursing as an overarching discipline (Julie 2014a:16–17).

**SL institutionalisation** refers to the process that perceives and supports SL as an essential component of the undergraduate nursing curriculum through embedding SL in the organisational structures and culture of the SoN. Tangible commitment to mainstream SL is demonstrated by a quality control system that measures SL indicators at the academic programme level to ensure that SL becomes an integral part of the infrastructure and everyday operations of the academic programmes and scholarly outputs of the SoN (Julie, Adejumo & Frantz 2015:2).

#### Contribution to field

This study makes a contribution to nursing education and SL because of the comprehensive nature of the SL implementation framework. It takes into account the factors needed to institutionalise SL in a nursing programme from an SL scholarship perspective.

### Literature review

The large-scale, systematic assessment of SL programmes in the USA could be linked to contestation of SL as a distinctive field of knowledge (Butin 2006b:473–475; Stanton & Erasmus 2013:61). This culminated in the identification of organisational factors regarded as crucial to the successful institutionalisation of SL (Brukardt *et al.* 2004:6; Furco 2002). The diverse frameworks that have been developed are informed by the particular ethos and philosophy reflected in the engagement of an HEI with the community it serves (Kasworm 2014:121). However, the hallmark characteristics for engagement advocated by Bringle and Hatcher (2011:411) are widely accepted by South African HEIs. Engagement activities undertaken by HEIs should therefore be scholarly. Such engaged scholarship activities should comprise teaching, research and service that embrace the processes and values of a civil democracy. These engaged scholarship activities should also be mutually beneficial to both the university and its community partners.

Julie (2014a:29) cites two widely used frameworks for SL institutionalisation: Furco's and the Proceedings of the Wingspread Conference (Brukardt *et al.* 2004). Furco (2002:3) has developed a self-assessment rubric for HEIs that measures the current level of SL institutionalisation according to three developmental stages on the horizontal axis against critical success factors for SL institutionalisation across five dimensions on the vertical axis. These dimensions are graded according to three stages to indicate at which level of SL institutionalisation an HEI is operating. At Stage 1, the critical mass building stage, an HEI is primarily focused on building a critical mass of SL scholars and developing SL activities across a campus. During Stage 2, the quality = building stage, institutional activities are focused on enhancing the quality rather than expanding the scope of SL programmes. Stage 3 focuses on sustaining SL by institutionalising SL in the core functions and operations of an HEI (Julie & Adejumo 2014:71).

Butin (2006b:477) regards Furco's framework (Furco 2002) as logical and incremental. Furco provides clear guidelines for operationalising the primary success factors for SL institutionalisation in a higher education environment against three developmental stages. However, Butin's main critique is that the framework does not take into account contextual differences (Julie 2014a:31).

## The Wingspread framework

The Wingspread framework locates the critical success factors for SL institutionalisation within the notions of engaged scholarship and institutional transformation. The following six factors have been identified as critical institutional success factors: (1) integration of engagement into operations; (2) forging partnerships as the overarching framework; (3) renewing and redefining discovery and scholarship; (4) integrating engagement into teaching and learning; (5) recruiting and supporting new champions, and (6) creating radical institutional change (Brukardt *et al.* 2004:6–15).

This researcher agrees with Butin (2006b:477) that this framework is not superior to Furco’s (2002) in terms of the implementation steps in general, in spite of its broader scoping (Julie & Adejumo 2014:33).

## The South African service-learning good practice guidelines

The process of formalising SL institutionalisation in higher education is playing out differently in South Africa because SL is perceived to be a policy imperative that directly relates to the transformation agenda of a democratic South Africa (Julie 2014a:33). Consequently, the structural and programme requirements that are essential to promoting and sustaining SL in academic programmes have been published within 5–7 years (HEQC 2006b:142) as opposed to the three decades the process took in the USA (Stanton & Erasmus 2013:61). The Institutional Audit Framework and Institutional Audit Criteria (HEQC 2004a:19) and the Good Practice Guide and Self-Evaluation Instruments for the Management of the Quality of Service-Learning (HEQC 2006b:138–145) form the foundations of the South African SL institutionalisation framework. This framework was informed by research conducted by the Community Higher Education Service Partnerships (CHESP) project (HEQC 2006a:18) and the SL institutionalisation work of Furco and Holland (2004) according to the HEQC (2006b:138, 143–144), as cited by Julie (2014a:34).

The following quality indicators have been identified: mission and philosophy; academic support for and involvement in SL development; institutional support for SL; student support and involvement in SL, and community participation and partnerships. These criteria are further classified into input, process, output and impact, and review factors for different levels, for example institution, faculty, school, programme and module (HEQC 2006a:21–31), as cited in (Julie 2014a:34). The HEQC regards the above indicators as good practice guidelines for SL.

Julie (2014a:37) agrees with Erasmus (2007:110) that some of the institutional indicators of the framework are ambiguously phrased, specifically indicators 4 and 7. The institutional input indicator 4 refers to ‘adequate resource allocation for delivering quality SL as part of the institution's [*sic*] core functions’ (HEQC 2006a:38). Similarly, the institutional process indicator 7 specifies that ‘there is adequate institutional support for the development and implementation of SL’ (HEQC 2006a:41). Although the expressed desire of the HEQC (2006a:7–8) not to be prescriptive is taken into consideration, the lack of clarity in these crucial institutional indicators adds to the quagmire of SL institutionalisation. A study of the mechanisms for institutionalising SL and community partner outcomes at 255 American universities concludes that the resource allocation strategies of universities play a definite role in the outcomes of SL (Stater & Fotheringham 2009:23).

Julie (2014a:38) commented that criticism is also levelled at the South African SL guidelines for the uncritical acceptance of the American-developed SL frameworks (Smith-Tolken & Williams 2011:5). In addition, the role of the individual does not receive proper attention in the South African framework, even though scholars agree that academics’ motivation is the decisive factor in successful SL implementation (Bender 2008:205; Erasmus 2007:112), possibly because it falls within the ambit of change management.

Consequently, Erasmus (2007:113–114), as a South African SL scholar, has developed a framework for understanding organisational behaviour for SL implementation based on the work of O’ Meara (2003:201–218). The five categories that Erasmus (2007:114) proposes as framing questions aim at understanding the behaviour of an organisation. The framing questions relate to the structure, human resources, politics and symbolism of SL institutionalisation with the intention of determining whether the organisational structures of an HEI support, incentivise and reward SL scholarship (Julie 2014a:40).

## The theoretical framework underpinning the study

The principles of organisational change in the theoretical model of Armenakis and Bedeian (1999:302) that informed the design of this design and development model of intervention research are described briefly (Julie 2014a:205–207) below.

**The change message should include a discrepancy that would convince the individual of the need to change:** The baseline survey completed in Phase 1 provided the evidence of the discrepancy and, hence, the impetus for collaboration during the research project. This contextual information about the status quo of SL scholarship amongst academics and SL practice in the undergraduate nursing programme at the SoN was linked to the national CE and SL policy brief for HEIs.

**The individuals should believe that they have the capability to change successfully:** The nurse educators were positively persuaded that they would be able to implement SL successfully at the operational level of the undergraduate nursing curriculum. They were guaranteed comprehensive support from the relevant university structures to effect the necessary changes in the undergraduate curriculum. This support was provided by the management of the SoN, the Community Engagement Unit and the Office of the Deputy Vice-Chancellor of UWC.

**The nurse educators should be convinced that it is in their best interests to change**: The assumption was that academics –who understood that developing socially responsive health professionals was a national imperative and that institutional audits would be conducted regularly by the HEQC – would commit as individuals to the SL institutionalisation process at UWC.

**The desired change is appropriate for the focal organisation:** The SoN should be confident that augmenting the dominant case-based teaching methodology with SL pedagogy would add value to the learning experiences of students.

## Research method and design

### Design

**FIGURE 1:**
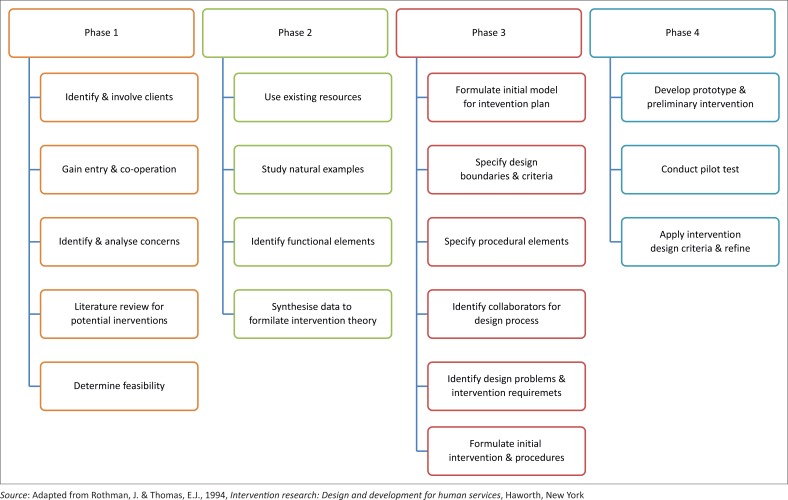
Executed operational steps of the design and development model.

This study was guided by the operational steps of the first four phases of the design and development intervention research model of Rothman and Thomas (1994:10–11) whilst using the mixed methods depicted in Figure 1 (Julie 2014a:53).

### Data collection procedure

Phase 1, problem analysis and project planning, provided the baseline information needed for the subsequent phases. The outcomes of Phase 2, information gathering and synthesis, identified and incorporated the functional elements from practice examples in the intervention theory for the study (Julie 2014a:202). During Phase 3, design, the intervention theory derived from the previous two phases culminated in the draft intervention plan for developing an SL implementation framework for the SoN (Julie 2014a:203). During Phase 4, early development and piloting, various prototypes were developed for the SL implementation framework. The researcher developed a questionnaire to measure the readiness and willingness of nurse educators to participate in the SL institutionalisation process; an SL module guide as a prototype of a SL pedagogical model; a contextualised SL definition for the SoN, and a monitoring and evaluation system for the SoN (Julie 2014a:204).

In addition, the SL module guide was piloted in a real setting with the fourth year undergraduate students in order to refine further the SL module development guidelines for the SL implementation framework (Julie 2014a:205).

### Context of the study

The SoN at the UWC is part of the Community and Health Science Faculty. This SoN is the largest residential nursing school in South Africa and has offered the Bachelor of Nursing degree since 2004 according to Jeggels, Traut and Africa (2013:2), as cited by Julie *et al.* (2015:4).

## Results

The results include the prescriptive elements and the domain boundaries for the SL implementation framework that were formulated for each of these elements.

## Prescriptive elements for the service-learning implementation framework

**FIGURE 2:**
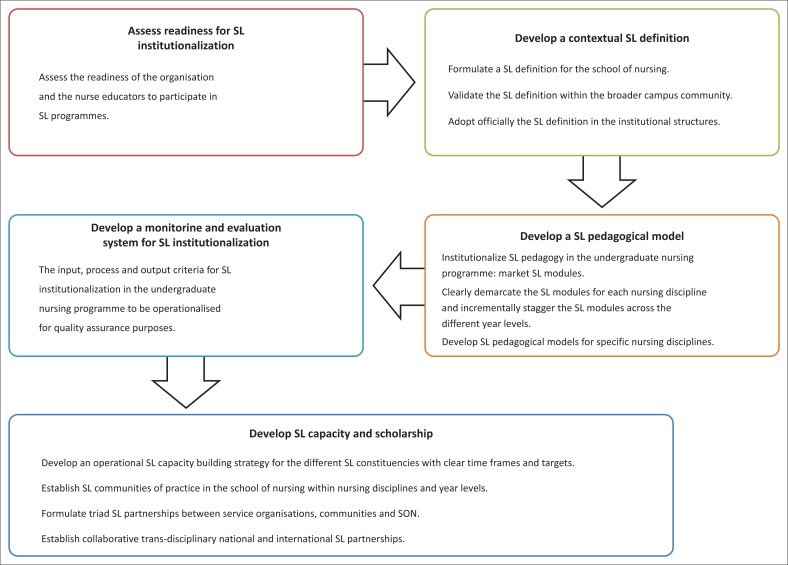
Prescriptive elements of the service-learning implementation framework.

The five prescriptive elements for the SL implementation framework (Figure 2) were formulated to bridge the gaps in the readiness for embedding SL in the undergraduate nursing programme, as identified by the core findings of Phases 1 and 2 (Julie 2014a:209). The key focal points aimed at (1) correcting the prevailing theory–practice gap that emanated from the conceptual confusion in the differentiation between SL and other forms of CE curricular activities (Julie 2014b:1832); (2) addressing the lack of knowledge relating to the national SL policy guidelines by involving academics; (3) capacity building and building SL scholarship in clinical supervisors (Julie *et al.* 2015:1); (4) developing an SL pedagogical model for the school by providing concrete implementation guidelines for embedding SL pedagogy in undergraduate nursing modules, and (5) formulating SL institutionalisation criteria for the nursing programme at the school in accordance with the SL quality indicators of the HEQC (Julie 2014a:211–218).

The available evidence of best practice models of SL institutionalisation supports the development of the above elements for successful SL institutionalisation.

## **Activities** of the service-learning implementation framework

**FIGURE 3:**
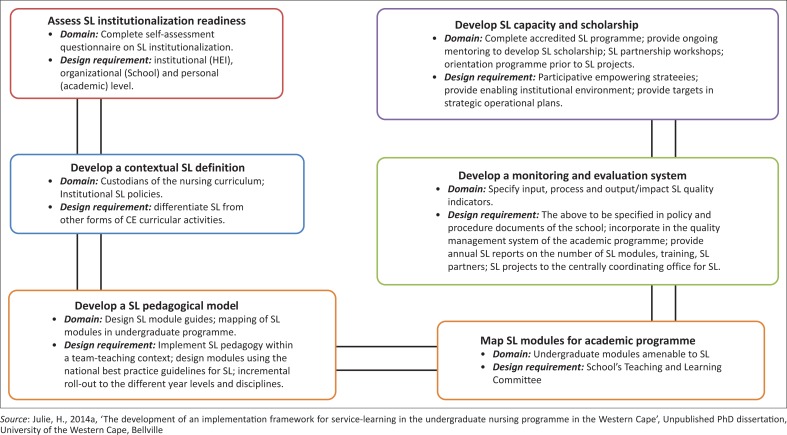
Activities of the service-learning implementation framework (Julie 2014).

The following activities of the SL implementation framework (Figure 3) were developed to provide the parameters for the prescriptive elements of the SL implementation framework identified in Figure 2.

A description of the structure of the framework and the essential issues that informed the final framework are highlighted in the discussion that follows.

## Discussion

The conceptual frameworks in Figures 2 and 3 provide a connection between readiness for SL institutionalisation, on the one hand, and theories of personal motivation and change management, on the other. Figure 3 illustrates the dependence of SL institutionalisation in an academic programme on the personal motivation of nurse educators to support the organisational change process implicit in SL institutionalisation. This framework further regards SL scholarship and personal willingness to participate in SL capacity-building activities as markers of change readiness for SL institutionalisation (Julie 2014a:210–215).

## Assessing service-learning institutionalisation readiness

Scholars in the field of organisational change advocate that organisational change processes should pay attention to the human factor (Self, Armenakis & Schraeder 2007:211). This resonates with the statement by O’ Meara (2003:202) that SL scholars should be supported both in their personal capacity and professionally whilst bearing in mind the structure, politics and culture of the organisation in which they work. Therefore, an individual's response to the proposed changes should receive close attention (Herold *et al.* 2008:343; Lamm & Gordon 2010:426) in order to mitigate the natural tendency of resisting change (Oreg 2003:680). It is thus important to establish whether the individuals who would be centrally involved in SL implementation are ready to effect the change (Todnem By 2005:375). This preparatory step should then lead to the empowering change strategies aimed at developing ‘ownership-taking behaviours’ within the individuals (Wright & Pandey 2010:77) as cited by Julie (2014b:1831).

## Developing a contextual service-learning definition

Although HEIs are encouraged to develop a contextual-specific definition that captures the unique ethos of an institution, the HEQC (2006b:25) stipulates that SL should (1) be a relevant and meaningful service to the community; (2) enhance academic learning indicating a clear connection between module objectives and service activities; (3) structure opportunities for reflection to transform, clarify, reinforce and expand concrete experiences into knowledge; (4) and demonstrate purposeful civic social responsibility. It is recommended that the contextual SL definition be developed through a consensus-seeking, democratic process such as the nominal group technique (Julie 2014b:1833).

## Developing service-learning capacity and service-learning scholarship

The framework (Figure 2) prioritises readiness and scholarship at the individual, communities of practice and organisational levels as critical components of SL institutionalisation (Julie 2014a:210). The author concurs with Butin (2006b:473) that SL should be positioned as scholarship, and Sandmann, Kiely and Grenier (2009:17) that the learning process should not be stifled by the traditional technical-rational approach to curriculum planning. SL theory is embedded in Dewey's notions of community, citizenship and democracy (Giles & Eyler 1994:78). These notions are pertinent to the principle of justice when developing collaborative community relations as stated by Rosner-Salazar (2003:64) that social justice is ’… having the perspective that allows one to take social action against social structural inequality and an understanding of oppression and equality which allows greater insight into methods of eradicating them’. This stance is supported by South African scholars (Erasmus 2007:109; Naudé 2012:74).

The SL implementation framework that was developed for the SoN (Figure 2) was based on the premise that true SL ‘cannot succeed without institutionalization’ (Shrader *et al.* 2008:29) and principles related to the organisational change theory of Armenakis and Bedeian (1999:302).

## The service-learning pedagogical model

The research took into account that the SL curriculum should be transformative in nature concerning the students’ personal, civic, moral and intellectual learning and development (Kiely 2005:7). The SL module guide that was developed as a SL pedagogical model for the undergraduate nursing programme incorporated these transformational curriculum elements in the five design criteria stipulated for SL modules by the HEQC (2006b:46–49). Hence, (1) the SL projects were embedded in the gender-based violence module outcomes, (2) the SL teaching strategy was clearly explained, (3) the SL project activities were contextualised in relation to the module content during lecturer contact periods and facilitated reflective sessions, (4) a detailed description of the SL requirements was integrated in the classroom-based activities, and (5) the assessment of the SL component was clarified (Boltman-Binkowski & Julie 2014:43).

Julie (2014b:38) states that the primary drivers for involvement in SL are motivation and the career stage of academics, according to O’ Meara (2003:202). Therefore, development of self-efficacy amongst the nurse educators at the SoN was prioritised because three of the five prescriptive elements of the implementation framework (Figure 2) related to this issue. It originated from the reasoning that these nurse educators’ insights into SL as a national imperative would provide the impetus for them to support SL institutionalisation in the undergraduate nursing programme at an individual level in their respective communities of practice across the various nursing disciplines (Todnem By 2005:375). The nurse educators at the SoN thus had to agree with the change message that SL pedagogy was a valuable and appropriate strategy to develop the social responsiveness of the graduates in the nursing programme. Since the SoN embraced team-teaching, key opinion makers were targeted as early adopters of SL in the undergraduate and postgraduate nursing programmes. The rationale being that their positions of authority would facilitate the embedding of SL pedagogy in the curriculum despite oppositional strategies expected from the late adopters (Julie 2014b:225). The personal valence of these drivers of the SL institutionalisation (Armenakis & Bedeian 1999:302) should be taken into account when designing structured SL capacity-building activities in order to minimise conflicting priorities between the SL institutionalisation targets set for the SoN and personal developmental goals (Julie 2014b:1832). Hence, the framework would ensure that sustained institutional support to the SoN is provided to institutionalise SL in the undergraduate curriculum, since a SL monitoring and evaluation system is an integral component of the framework (Julie 2014a:206).

## Monitoring and evaluation system for service-learning institutionalisation

Julie (2014a:211) states that the Institutional Audit Framework and Institutional Audit Criteria (HEQC 2004a:11, 19) and the Good Practice Guide and Self-evaluation Instruments for Managing the Quality of Service-Learning (HEQC 2006a:21–33) are used as the golden standard for the South African context. Hence, the guidelines for developing a monitoring and evaluation system for the school, the academic programme and the module levels rely heavily on the abovementioned documents.

The national institutionalisation indicators had been contextualised to the SoN for the SL institutionalisation indicators at the level of the school and the academic programme. The researcher proposes that a self-assessment of the school environment be undertaken as a strategy to create awareness amongst the staff members (Julie 2014a:212). Likewise, the self-assessment activity of the nursing programmes should be undertaken by the gatekeepers of the curriculum and the opinion makers at the school to provide the necessary rudimentary support for the mapping of SL modules in the academic programme via the input, process, output, and impact criteria for the academic programme as specified by the HEQC (2006a:47–49). The SL curricula also need to be properly sequenced for maximum educational benefits and the integration of SL into the curriculum involves a pedagogical strategy that goes beyond the scope of a single course (Osman & Petersen 2013:7).

## Module-level guidelines

This assessment or monitoring activity should be undertaken by the teaching teams under the guidance of a CE scholar and/or an experienced SL champion with the aim of broadening the base of SL scholarship at the school. People regarded as influential in the communities of practice at the school and the HEI should be motivated to become drivers of the SL institutionalisation process in order to provide the necessary mentoring to the early adopters of SL (Julie 2014a:215–216).

The SL implementation framework satisfies the goals of SL institutionalisation, namely to (1) develop a common language, (2) compile a set of principles to guide practice and (3) ensure the allocation of resources to facilitate the SL teaching methodology (HEQC 2006a:138). The contextualised SL definition that was formulated for the SoN contributes to the SL operationalisation discourse at the HEI (Julie 2014a:219).

## Ethical considerations

The intervention study met all the prescribed ethical procedures of the UWC and received ethical clearance from the Senate Ethics Committee, project registration number 11/1/37 (Julie 2014b:1836).

## Limitations of the study

Limited success was achieved with the building of an authentic community of practice amongst the SL teaching team during the piloting phase of the SL pedagogical model.

## Recommendations

The developed implementation framework needs to be implemented and evaluated as the next steps to complete the Design & Development Intervention Research. The SL definition that was developed for the school should be regarded as a work in progress, since it was developed before the 11 nurse educators completed the accredited SL short course in 2013. Therefore, this preliminary SL definition will be further refined by a master's degree nurse educator student who is prepared to take up the challenge.

## Conclusion

This study addresses the gap identified that most HEIs in South Africa fail to establish a standard practice for SL within the formalised systems of their respective academic programmes. The SL implementation framework of this study specifies the intervention elements (change strategies) needed to bridge the gaps that have been identified by the core findings of Phases 1 and 2. The design phase thus includes change intervention elements aimed at bridging the prevailing theory-practice gap that emanated from the conceptual confusion relating to (1) differentiating between SL and other forms of community engagement curricular activities, (2) addressing the lack of knowledge of the national SL policy guidelines by involving the academic staff and clinical supervisors in SL capacity building and SL scholarship, (3) developing an SL pedagogical model for the SoN by providing concrete implementation guidelines to embed SL pedagogy in undergraduate nursing modules that are amenable to this pedagogy , and (4) formulating SL institutionalisation criteria for the nursing programme at the SoN in accordance with the SL quality indicators of the HEQC.
